# Identification of genes and pathways in nasopharyngeal carcinoma by bioinformatics analysis

**DOI:** 10.18632/oncotarget.19478

**Published:** 2017-07-22

**Authors:** Fang Chen, Congxiang Shen, Xiaoqi Wang, Huigang Wang, Yanhui Liu, Chaosheng Yu, Jieyu lv, Jingjing He, Zhong Wen

**Affiliations:** ^1^ Department of Otorhinolaryngology-Head and Neck Surgery, Zhujiang Hospital, Southern Medical University, Guangzhou, China; ^2^ Department of Otorhinolaryngology-Head and Neck Surgery, The Second Affiliated Hospital of Xinjiang Medical University, Xinjiang, China; ^3^ Department of Otorhinolaryngology-Head and Neck Surgery, Guangzhou Red Cross Hospital, Medical College, Jinan University, Guangzhou, China; ^4^ Department of Otorhinolaryngology-Head and Neck Surgery, Jiangmen Central Hospital, Jiangmen, China; ^5^ Department of Otorhinolaryngology-Head and Neck Surgery, The First Affiliated Hospital of Xiamen University, Xiamen, China

**Keywords:** nasopharyngeal carcinoma, differentially expressed genes, functional enrichment analysis, protein–protein interaction (PPI) network, hub genes

## Abstract

Nasopharyngeal carcinoma is a metastatic malignant tumor originating from nasopharyngeal epithelium. Lacking or nonspecific symptoms of patients with early stage nasopharyngeal carcinoma have significantly reduced the accuracy of diagnosing and predicting nasopharyngeal carcinoma development. This study aimed to identify gene signatures of nasopharyngeal carcinoma and uncover potential mechanisms. Two gene expression profiles (GSE12452 and GSE13597) containing 56 nasopharyngeal carcinoma samples and 13 normal control samples were analyzed to identify the differentially expressed genes. In total, 179 up-regulated genes and 238 down-regulated genes were identified. Functional and pathway enrichment analysis showed that up-regulated genes were significantly involved in cell cycle, oocyte meiosis, DNA replication and p53 signaling pathway, while down-regulated genes were enriched in Huntington's disease,metabolic pathways. Subsequently, the top 10 hub genes, *TOP2A* (topoisomerase (DNA) II alpha), *CDK1* (cyclin-dependent kinase 1), *CCNB1* (cyclin B1), *PCNA* (proliferating cell nuclear antigen), *MAD2L1* (mitotic arrest deficient 2 like 1), *BUB1* (budding uninhibited by benzimidazoles 1 homolog), *CCNB2* (cyclin B2), *AURKA* (aurora kinase A), *CCNA2* (cyclin A2), *CDC6* (cell division cycle 6 homolog), were identified from protein-protein interaction network. Furthermore, Module analysis revealed that the ten hub genes except *TOP2A* were belonged to module 1, indicating the upregulation of these hub genes associated molecular pathways in nasopharyngeal carcinoma might activate nasopharyngeal carcinoma pathogenesis. In conclusion, this study indicated that the identified differentially expressed genes and hub genes enrich our understanding of the molecular mechanisms of nasopharyngeal carcinoma, which could eventually translate into additional biomarkers to facilitate the early diagnosis and therapeutic approaches.

## INTRODUCTION

Nasopharyngeal carcinoma (NPC) is the most common squamous cell carcinoma arising from nasopharynx. Worldwide, nasopharyngeal carcinoma is predominant in east and southeast parts of Asia, south-central Asia, and north and east Africa [1]. In addition, the incidence of NPC in western countries is less than 1/100,000 while it is highly prevalent with an incidence of 20/100,000 in China, and the new NPC case increases exponentially as sixty thousand new NPC cases were reported in 2015 [2]. Furthermore, the symptoms of patients with early stage disease are often lacking or nonspecific. Therefore, clinically NPC is often diagnosed in a late stage and has a relatively poor survival rate after diagnosis [3]. Epstein-Barr virus (EBV) infection has a close correlation with NPC development, and patients with NPC have a distinct anti-EBV antibody profile, especially immunoglobulin A (IgA) antibodies [4]. The plasma EBV DNA load may improve the accuracy of diagnosing NPC in high-risk individuals, but it appears to have limited value in screening patients who have early stage NPC and predicting NPC development [3]. In addition, increasing evidence proves that polygenes and cell pathways are involved in the development and progression of NPC [5]. So far, the precise molecular mechanisms for the development of nasopharyngeal cancer are unknown, which limits the potential for early diagnosis and treatment of NPC. Therefore, it is crucial to investigate the molecular mechanisms in nasopharyngeal carcinoma progression and discover additional biomarkers to facilitate the early diagnosis and curative treatment.

In recent years, the high-throughput platforms for analysis of gene expression, such as microarray technologies, has been broadly used to obtain general genetic alteration during tumorigenesis [6, 7]. Many gene expression analysis of nasopharyngeal carcinoma involved microarray technology and identified many differentially expressed genes (DEGs) in pathways, molecular functions or biological process according to the literature. However, comparative analysis of DEGs in independent studies shows that there is little overlap, and there is no reliable biomarker to identify nasopharyngeal carcinoma tissues from normal tissues. The bioinformatics analysis is essential to process the large amounts of data generated by microarray technology. However, despite this progress, the interactions among DEGs and the pathways in the interaction network remain unclear. In this study, we analyzed two mRNA microarray dataset to obtain DEGs between nasopharyngeal carcinoma tissues and normal tissues samples. Furthermore, the key genes and pathways associated with nasopharyngeal carcinoma were identified by functional enrichment and network analysis of identified DEGs. Our results suggested that data mining and integration could be a useful method to predict progression of nasopharyngeal carcinoma, to understand the mechanism of the occurrence and development of tumor, and eventually to facilitate the early diagnosis and therapeutic approaches through identifying additional biomarkers. With the aid of analyzing their biological functions and pathways, we may light the further insight of NPC development at molecular level and explored the potential candidate biomarkers for diagnosis, drug targets and prognosis.

## RESULTS

### Identification of DEGs

**Figure 1:**
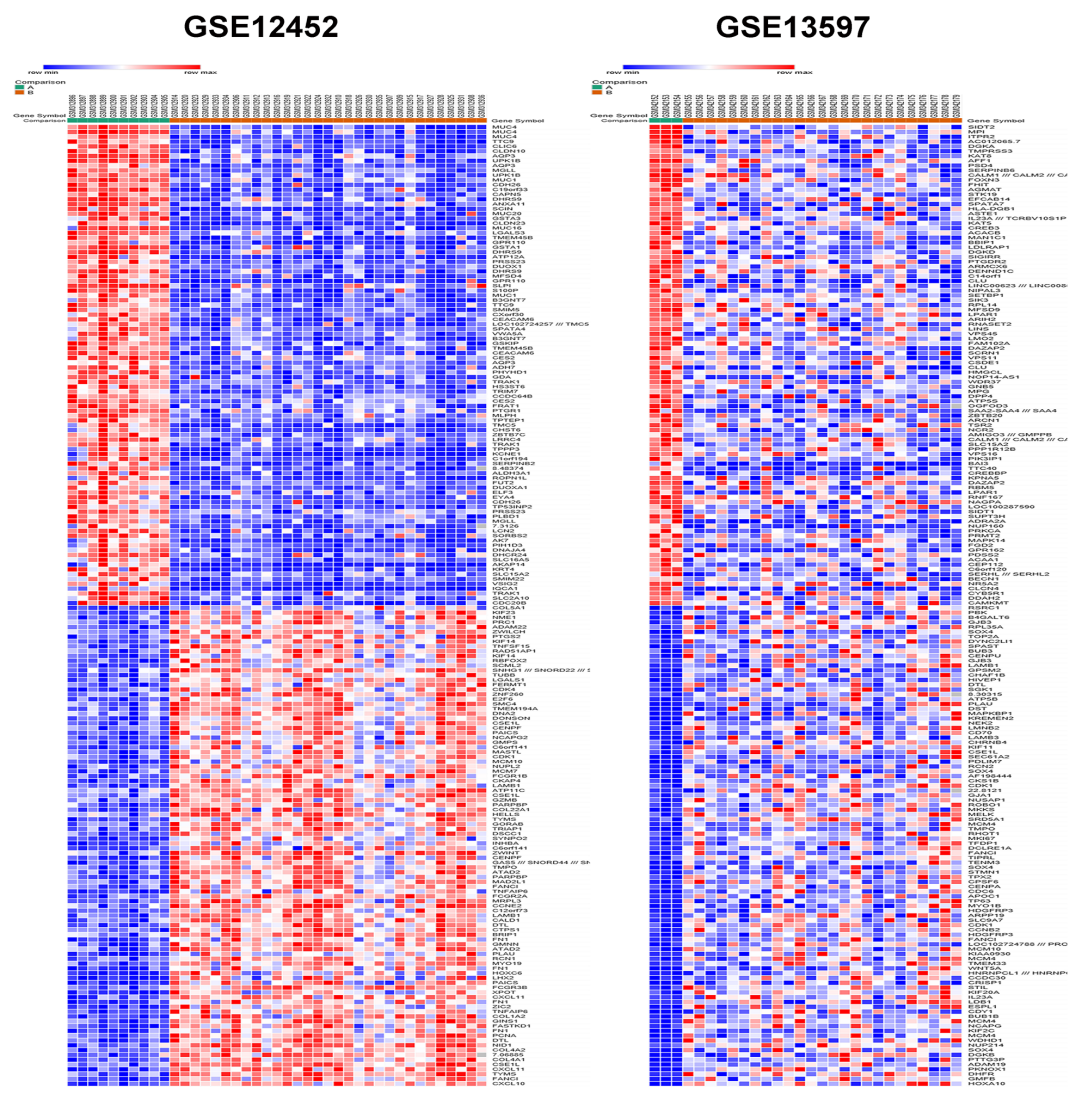
Heat map of the top 200 differentially expressed genes of GSE12452 and GSE13597 (100 up-regulated genes and 100 down-regulated genes) Red: up-regulation; blue: down-regulation

The raw data file of GSE12452 and GSE13597 were uploaded to GEO2R (http://www.ncbi.nlm.nih.gov/geo/geo2r/) to screen differentially expressed genes (DGEs) between nasopharyngeal carcinoma and normal samples. A total number of 56 nasopharyngeal carcinoma samples and 13 normal control samples were analyzed. The top 250 DEGs were respectively screened out in GSE12452 and GSE13597 datasets based on GEO2R. Of which, 417 DEGs lists were finally identified using FunRich_V3 software [8], consisting of 179 up-regulated genes and 238 down-regulated genes in nasopharyngeal carcinoma samples compared to normal nasopharyngeal tissue samples. DEGs expression heat maps (top 100 up-regulated and top 100 down-regulate genes) of GSE12452 and GSE13597 datasets respectively constructed by a new web-based tool Morpheus (https://software.broadinstitute.org/morpheus/) were depicted in Figure 1. These identified up-regulated and down-regulate genes were used for functional and pathway enrichment analysis.

### GO term and KEGG pathway enrichment analysis

Accumulating evidence suggests that co-expression genes consists of a group of genes with similar expression profiles and participates in parallel biological processes. The up-regulated genes and down-regulated genes were respectively uploaded to DAVID to gain the functional annotation and pathway enrichment of identified DEGs. The top five terms enriched in each category were selected according to P value. The up-regulated genes were significantly involved in biological processes associated cell division, DNA replication, mitotic nuclear division, G1/S transition of mitotic cell cycle and cell proliferation, while the down-regulated genes were mainly enriched in cilium movement, motile cilium assembly, cilium-dependent cell motility, inner and outer dynein arm assembly (Table 1). GO cellular component (CC) analysis showed that the up-regulated DEGs were significantly enriched in nucleoplasm and cytosol and the down-regulated DEGs were enriched in axoneme and cilium. In addition, the molecular function of up-regulated DEGs were mainly associated with protein binding, ATP binding, and ATPase activity, while the down-regulated DEGs were involved in microtubule motor activity and serine-type endopeptidase inhibitor activity (Table 1).

**Table 1:** Gene ontology analysis of differentially expressed genes associated with NPC^a^

Category	Term	Count	%	P Value
**Up-regulated**				
	GO:0051301∼cell division	27	16.0	7.2E-16
	GO:0006260∼DNA replication	18	10.7	1.8E-13
GOTERM_BP_DIRECT	GO:0007067∼mitotic nuclear division	21	12.4	4.2E-13
	GO:0000082∼G1/S transition of mitotic cell cycle	13	7.7	3.2E-10
	GO:0008283∼cell proliferation	19	11.2	2.0E-08
	GO:0005654∼nucleoplasm	65	38.5	3.9E-13
	GO:0005634∼nucleus	87	51.5	4.0E-09
GOTERM_CC_DIRECT	GO:0005829∼cytosol	63	37.3	6.8E-09
	GO:0000777∼condensed chromosome kinetochore	10	5.9	1.1E-07
	GO:0030496∼midbody	11	6.5	3.2E-07
	GO:0005515∼protein binding	119	70.4	7.7E-08
	GO:0005524∼ATP binding	37	21.9	2.2E-07
GOTERM_MF_DIRECT	GO:0016887∼ATPase activity	10	5.9	7.3E-05
	GO:0008017∼microtubule binding	9	5.3	9.5E-04
	GO:0019901∼protein kinase binding	12	7.1	1.1E-03
**Down-regulated**				
	GO:0003341∼cilium movement	7	3.0	2.1E-07
	GO:0044458∼motile cilium assembly	5	2.1	1.2E-05
GOTERM_BP_DIRECT	GO:0060285∼cilium-dependent cell motility	4	1.7	1.5E-04
	GO:0036159∼inner dynein arm assembly	4	1.7	2.5E-04
	GO:0036158∼outer dynein arm assembly	4	1.7	4.0E-04
	GO:0005930∼axoneme	11	4.7	1.1E-08
	GO:0005858∼axonemal dynein complex	5	2.1	2.3E-06
GOTERM_CC_DIRECT	GO:0005929∼cilium	10	4.3	3.1E-05
	GO:0005874∼microtubule	13	5.6	1.1E-04
	GO:0005737∼cytoplasm	76	32.5	9.8E-04
	GO:0003777∼microtubule motor activity	7	3.0	1.3E-04
	GO:0004867∼serine-type endopeptidase inhibitor activity	6	2.6	2.6E-03
GOTERM_MF_DIRECT	GO:0003779∼actin binding	9	3.8	6.1E-03
	GO:0051087∼chaperone binding	5	2.1	8.1E-03
	GO:0003774∼motor activity	4	1.7	0.027

^a^ If there were more than five terms enriched in this category, top five terms were selected according to P value. Count: the number of enriched genes in each term.

Moreover, the significantly enriched KEGG pathways of the up-regulated DEGs and down-regulated DEGs were displayed in Table 2. Fifteen KEGG pathways were over-represented in up-regulated DGEs, including cell cycle, p53 signaling pathway, DNA replication, small cell lung cancer, ECM-receptor interaction and pathways in cancer. Five KEGG pathways were significantly enriched in Huntington's disease,metabolic pathways, drug metabolism-cytochrome P450, tight junction and tyrosine metabolism for the down-regulated DEGs (Table 2). The most significantly enriched GO terms and KEGG pathways of up-regulated genes and down-regulated genes allowed us to better understand the interactions of DEGs at functional level.

**Table 2:** KEGG pathway analysis of differentially expressed genes associated with NPC

Term	Count	%	P Value	Genes
**Up-regulated**				
hsa04110: Cell cycle	20	11.8	1.94E-16	*CDC6, CDK1, SKP2, TTK, CHEK1, ESPL1, PTTG1, MCM4, CCNB1, CCNE2, MAD2L1, MCM7, CCNB2, BUB1, PCNA, BUB1B, MDM2, GADD45A, CCNA2, BUB3*
hsa04115: p53 signaling pathway	10	5.9	9.04E-08	*BID, CCNB1, CCNE2, CDK1, CCNB2, RRM2, MDM2, CHEK1, PMAIP1, GADD45A*
hsa03030: DNA replication	7	4.1	3.92E-06	*RFC5, RFC3, MCM7, POLD1, POLE, PCNA, MCM4*
hsa05222: Small cell lung cancer	9	5.3	7.66E-06	*CCNE2, CKS1B, COL4A2, COL4A1, ITGAV, CKS2, SKP2, LAMB1, FN1*
hsa04512: ECM-receptor interaction	7	4.1	6.20E-04	*COL4A2, COL4A1, ITGAV, HSPG2, LAMB1, FN1, HMMR*
hsa05200: Pathways in cancer	14	8.3	8.32E-04	*BID, WNT5A, CKS1B, COL4A2, COL4A1, SKP2, BIRC5, MECOM, CCNE2, ITGAV, CKS2, MDM2, LAMB1, FN1*
hsa04114: Oocyte meiosis	7	4.1	2.0E-03	*CCNE2, CDK1, MAD2L1, BUB1, AURKA, ESPL1, PTTG1*
hsa03420: Nucleotide excision repair	5	3.0	2.4E-03	*RFC5, RFC3, POLD1, POLE, PCNA*
hsa03430: Mismatch repair	4	2.4	2.6E-03	*RFC5, RFC3, POLD1, PCNA*
hsa04914: Progesterone- mediated oocyte maturation	6	3.6	4.0E-03	*CCNB1, CDK1, MAD2L1, CCNB2, BUB1, CCNA2*
hsa05166: HTLV-I infection	9	5.3	0.012	*WNT5A, MAD2L1, POLD1, POLE, PCNA, BUB1B, CHEK1, PTTG1, BUB3*
hsa04068: FoxO signaling pathway	6	3.6	0.023	*CCNB1, SGK1, CCNB2, SKP2, MDM2, GADD45A*
hsa00670: One carbon pool by folate	3	1.8	0.024	*TYMS, SHMT2, DHFR*
hsa00240: Pyrimidine metabolism	5	3.0	0.037	*TYMS, UMPS, POLD1, RRM2, POLE*
hsa05203: Viral carcinogenesis	7	4.1	0.038	*CCNE2, CDK1, SKP2, MDM2, CHEK1, PMAIP1, CCNA2*
**Down-regulated**				
hsa05016: Huntington's disease	8	3.4	2.0E-03	*DNAH9, DNALI1, DNAI1, CREBBP, DNAH3, DNAH2, DNAH7, DNAH6*
hsa01100: Metabolic pathways	20	8.5	0.016	*GDA, ADSSL1, GMDS, GNE, DHRS9, ADH7, COMT, CDS1, AK7, AGMAT, MAN1C1, ALDH3A1, DGKA, ALOX15, ATP6V0E2, GCK, FUT3, MGLL, FUT2, ATP6V0A4*
hsa00982: Drug metabolism–cytochrome P450	4	1.7	0.026	*FMO5, GSTA3, ADH7, ALDH3A1*
hsa04530: Tight junction	5	2.1	0.040	*PRKCA, CGN, MYH11, CLDN10, CLDN23*
hsa00350: Tyrosine metabolism	3	1.3	0.042	*ADH7, COMT, ALDH3A1*

### PPI network construction and modules selection

**Figure 2:**
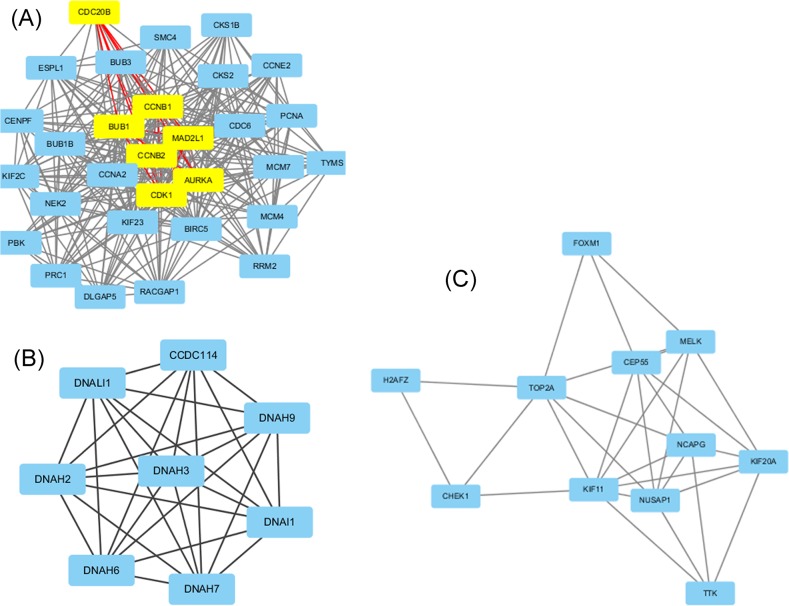
Top 3 modules from the protein-protein interaction network according to MCODE score **(A)** module 1, yellow nodes belong to one sub-module, while light blue nodes belong to the other sub-module. **(B)** module 2, **(C)** module 3.

The protein-protein interaction (PPI) network provides valuable information for understanding cellular functions and biological processes. The top 10 hub nodes with higher degrees were screened out from the PPI network of DEGs consisted of 374 nodes and 1218 edges based on the information in the STRING database. These hub genes included *TOP2A* (topoisomerase (DNA) II alpha), *CDK1* (Cyclin-dependent kinase 1), *CCNB1* (cyclin B1), *PCNA* (proliferating cell nuclear antigen), *MAD2L1* (mitotic arrest deficient 2 like 1), *BUB1* (budding uninhibited by benzimidazoles 1 homolog), *CCNB2* (Cyclin B2), *AURKA* (Aurora kinase A), *CCNA2* (Cyclin A2), *CDC6* (cell division cycle 6 homolog). Among these genes, *TOP2A* and *CDK1* showed higher node degrees, which were respectively 66 and 61. In addition, the top three significant modules were obtained from PPI network of DEGs using MCODE analyzing (Figure 2). Among which module 1 including 30 nodes and 299 edges could be further divided into two sub-modules using MCODE analyzing, One included 7 nodes and 21 edges and the other included 23 nodes and 134 edges (Figure 2a). Furthermore, Functional and pathway enrichment analysis of the genes in these three modules were performed by DAVID (Table 3). Results revealed that genes in module 1 and module 3 were mainly associated with cell cycle, cell division, oocyte meiosis and p53 signaling pathway while genes in module 2 were mainly enriched in cilium or flagellum-dependent cell motility, axonemal dynein complex assembly and Huntington's disease. Moreover, the top ten hub genes expect *TOP2A* were belonged to module 1, which together determined the key pathways associated with nasopharyngeal carcinoma. Through the construction of PPI network and selection module, we screened 10 hub key genes, which can provide new ideas for the treatment of nasopharyngeal carcinoma.

**Table 3:** Functional and pathway enrichment analysis of the genes in modules ^a^

Pathway description	Count	FRD	Nodes
**Module 1**			
GO 0044772: mitotic cell cycle phase transition	11	1.7E-14	*AURKA, BIRC5, BUB1B, CCNB1, CCNB2, CDC6, CDK1, CENPF, NEK2, PCNA, TYMS*
GO 0051301: cell division	11	8.0E-13	*AURKA, BIRC5, BUB1, BUB1B, CCNB1, CCNB2, CDC6, CDK1, CENPF, NEK2, SMC4*
GO 0007067: mitotic nuclear division	9	3.9E-10	*BIRC5, BUB1, CCNB1, CCNB2, CDC6, CDK1, CENPF, DLGAP5, SMC4*
GO 0007088: regulation of mitotic nuclear division	7	1.9E-09	*AURKA, BIRC5, BUB1, CDC6, CENPF, DLGAP5, NEK2*
GO 0000086: G2/M transition of mitotic cell cycle	7	3.3E-09	*AURKA, BIRC5, CCNB1, CCNB2, CDK1, CENPF, NEK2*
hsa04110: Cell cycle	6	3.1E-08	*BUB1, BUB1B, CCNB1, CCNB2, CDC6, PCNA*
hsa04114: Oocyte meiosis	4	9.1E-05	*AURKA, BUB1, CCNB1, CCNB2*
hsa04914: Progesterone-mediated oocyte maturation	3	1.9E-03	*BUB1, CCNB1, CCNB2*
hsa04115: p53 signaling pathway	5	0.028	*CCNE2, CCNB1, CDK1, CCNB2, RRM2*
**Module 2**			
GO 0007018: microtubule-based movement	7	3.1E-10	*CCDC114, DNAH2, DNAH3, DNAH6, DNAH7, DNAH9, DNAI1*
GO 0001539: cilium or flagellum-dependent cell motility	4	1.4E-08	*DNAH2, DNAH3, DNAH6, DNAH7*
GO 0070286: axonemal dynein complex assembly	3	2.1E-04	*CCDC114, DNAH7, DNAI1*
GO 0003341: cilium movement	3	7.7E-04	*CCDC114, DNAH7, DNAI1*
GO 0035082: axoneme assembly	3	1.1E-03	*CCDC114, DNAH7, DNAI1*
hsa05016: Huntington's disease	4	1.2E-04	*DNAH2, DNAH3, DNAI1, DNALI1*
**Module 3**			
GO 1903047: mitotic cell cycle process	9	3.8E-08	*CEP55, CHEK1, FOXM1, KIF20A, MELK, NCAPG, NUSAP1, TOP2A, TTK*
GO 0000278: mitotic cell cycle	9	5.1E-08	*CEP55, CHEK1, FOXM1, KIF20A, MELK, NCAPG, NUSAP1, TOP2A, TTK*
GO 0007059: chromosome segregation	5	1.1E-04	*KIF11, NCAPG, NUSAP1, TOP2A, TTK*
GO 0051301: cell division	6	1.6E-04	*CEP55, KIF11, KIF20A, NCAPG, NUSAP1, TOP2A*
GO 0022402: cell cycle process	7	3.8E-04	*CEP55, FOXM1, KIF20A, MELK, NCAPG, NUSAP1, TOP2A*

^a^ If there were more than five terms enriched in this category, top five terms were selected according to P value. Count: the number of enriched genes in each term.

## DISCUSSION

Despite advances in radiation technology, distant metastasis was still the major pattern of treatment failure of NPC. More effective treatment modalities to reduce the rate of distant metastasis attract tremendous attentions [9]. Therefore, understanding of the etiological factors and mechanisms of NPC progression are essential to improve survival rate and prevention. Recently, microarray technology has been rapidly developed and been widely used in progression of diseases, which promotes the identification of targets for diagnosis, therapeutic, and prognosis of tumors [10]. In this study, a total of 417 DEGs were screened out in GSE12452 and GSE13597 datasets, consisting of 179 up-regulated genes and 238 down-regulated genes. Function annotation and KEGG pathway enrichment analysis showed that these up-regulated genes were mainly involved in cell division, cell cycle, DNA replication, cell proliferation and p53 signaling pathway, while the down-regulated genes were mainly enriched in cilium movement, motile cilium assembly, Huntington's disease, inner and outer dynein arm assembly. Recent studies indicated that single nucleotide polymorphisms in genes of cell cycle pathway and NF-κB pathway can potentially predict the clinical responses to radiotherapy for NPC patients [11]. These results were consistent with the fact that cancer development and progression are closely related to the defective function of cell cycle and cell proliferation regulators. Moreover, previous research demonstrated that the downregulation of mitochondrial Ca (2+) signaling has crucial role in triggering cell death via pathological Ca (2+) overload [12]. Therefore, monitoring these signaling pathways may aid prediction of NPC progression.

Based on the PPI network, 10 hub genes that can highlight the further insight of NPC development at molecular level for therapeutic studies were obtained. *TOP2A* (topoisomerase (DNA) II alpha), a nuclear enzyme which is involved in cell division and cell cycle, was identified as one of the hub genes exhibiting the highest degree of connectivity in current study. Kaplan et al. [13] demonstrated that *TOP2A* represented a direct molecular target of anthracyclin-based chemotherapy and topoisomerase inhibitor, such as etoposide. It has also reported that *TOP2A* was the well-known good prognostic marker in breast cancers, which was associated with a favorable response to anthracyclin-based therapy [14–16]. Moreover, Lan et al. [17] reported that the overexpression of *TOP2A* significantly correlated with more advanced American Joint of Cancer Committee (AJCC) stages and independently predicted worse disease-specific survival (DSS) and distant metastasis-free survival (DMFS) in nasopharyngeal carcinoma. In addition, *TOP2A* overexpression was also reported in other cancer types, such as prostate cancer [18], endometrial cancer [19], colorectal [20], and lung carcinomas [21], and so on. These results suggested that *TOP2A* in this study was identified as one of the top-ranking upregulated candidates among the differentially expressed genes in NPC tissues, which is demonstrated by previous research. The second hub gene cyclin-dependent kinase 1 (*CDK1*) that controlling cell cycle events including DNA replication and segregation, transcriptional programs and cell morphogenesis have been identified. It was suggested that *CDK1* played a significant role in the control of the eukaryotic cell cycle by restricting the centrosome cycle as well as mitotic onset [22]. In addition, Lactate dehydrogenase (LDH) inhibition by oxamate induced G2/M cell cycle arrest via downregulation of the *CDK1*/cyclin B1 pathway might serve as a promising therapeutic target for NPC treatment [23]. Moreover, the involvement of *CDK1* in tumorgenesis was postulated in various types of cancer, including laryngeal cancer [24] and ovarian cancer cells [25]. Increased levels of cyclin B1 (*CCNB1*) could activate *CDK1*, which controlled key early mitotic events including growth inhibition and S-G2/M phase arrest [26]. Tulalamba et al. [27] reported that cyclin B1 was accountable for cell cycle progression at G2/M and G1/S phase induced in NPC compared to those in non-cancerous cells. Accumulating evidence showed that the suppression of cyclin B1 expression by curcumin resulted in G2/M arrest in a NPC cell line [28]. Overexpression of *CCNB1* was found in many different diseases, including colorectal cancer, breast, pancreatic cancer, and meningioma [29–32]. Overexpression of *CCNB1* was also found in our study, which suggested that the upregulation of *CDK1*/cyclin B1 pathway might activate cell cycle progression in NPC.

The other two members of cyclin family cyclin A2 (*CCNA2*) and cyclin B2 (*CCNB2*) that related to cell cycle at the G2/M (mitosis) transition and affected chromosomal stability were identified. It was reported that Cyclin A2 (*CCNA2*) was significantly overexpressed in various cancer types including ER+ breast cancer [33], splenic diffuse red pulp small B-cell lymphoma [34], rectal neuroendocrine tumors [35]. Wu et al. [36] reported that MicroRNA-188 exerted anticancer effects in human nasopharyngeal carcinoma (NPC) via downregulation of multiple G1/S related cyclin/*CDKs* including *CCNA2* and Rb/E2F signaling pathway. And Huang et al. [37] reported that *CCNB2* tuned G2/M transitions time though regulating a Golgi checkpoint. However, cyclin B2 overexpression was a poor prognostic biomarker in non-small cell lung cancer [38], bladder cancer [39], invasive breast carcinoma [40], colorectal adenocarcinoma [41]. In the present study, the three identified cyclin family members were overexpressed and had close interaction, indicating the joint function in human nasopharyngeal carcinoma.

Expression of the proliferating cell nuclear antigen (*PCNA*) genes has an essential role in the control of eukaryotic DNA replication by increasing the polymerase's processibility during elongation of the leasing strand. Recent evidence has demonstrated that *PCNA* was a key factor in cell cycle regulation and DNA replication via interacting with cell cycle-regulated proteins [42, 43]. In addition, cell cycle-regulated proteins including *PCNA* were found to be prognostic and diagnostic implications of salivary gland cancers [44]. However, Wang's research [45] showed that overexpression of *PCNA* was not a useful marker for advanced NPC, which did not predict the results of treatment in patients. For aurora kinase A (*AURKA*), one of mitotic serine/threonine kinases contributes to the regulation of cell cycle progression, which associates with centrosome and the spindle microtubules during mitosis and plays a critical role in various mitotic events including the establishment of mitotic spindle, centrosome separation as well as maturation, formation and function of the bipolar spindle, and cytokinesis. Genetic amplification and mRNA and protein overexpression of *AURKA* are common in many different tumors, which have allowed aurora kinase A to be a potential target for development of cancer therapeutics in early-phase clinical trials [46].

Cell division cycle 6 homolog (*CDC6*) is involved in the initiation of DNA replication and participates in checkpoint controls that ensure DNA replication is completed before mitosis is initiated. Huang et al. [47] reported that DNA replication initiator *CDC6* also activated ribosomal DNA transcription initiation during cell cyle at G1/S (start) transition. High expression of CDC6 in epithelial ovarian cancer (EOC) cells was indicated as a new potential therapeutic target for EOC patients [48]. In addition, *CDC6* as a key regulatory target for tumorgenesis was postulated in various types of cancer including prostate cancer [49], neuroblastoma cell [50], hepatocellular carcinoma [51]. However, the biological function and clinical significance of *CDC6* in NPC remain unclear. In this study, serine/threonine-protein kinase *BUB1*(budding uninhibited by benzimidazoles 1 homolog) that performs two crucial functions during mitosis (spindle-assembly checkpoint signaling and correct chromosome alignment) was identified as one of the hub genes. Moreover, mitotic arrest deficient 2 like 1 (*MAD2L1*) that is required for the execution of the mitotic checkpoint which monitors the process of kinetochore-spindle attachment and inhibits of the anaphase promoting complex was also identified as hub gene. Taken together, these data suggested that the hub genes overexpressed and highly connected may be involved in the regulation of mitosis, and cell cycle process of NPC via the checkpoint mechanism [52, 53].

Module analysis of the PPI network revealed the top ten hub genes identified expect *TOP2A* were belonged to module 1, indicating that these hub genes had close interaction and together determined the key pathway associated with nasopharyngeal carcinoma. Functional and pathway enrichment analysis of genes in module 1 showed that the development of NPC was mainly involved in cell cycle, Oocyte meiosis, Progesterone-mediated oocyte maturation and p53 signaling pathway. Altogether, we propose that upregulation of these molecular pathways in NPC might play a role in the NPC pathogenesis, which could eventually translate into additional biomarkers to facilitate the early diagnosis and therapeutic approaches.

In conclusion, the current study was intended to identify DEGs with integrated bioinformatics analysis to find the potential biomarkers and predict progression of nasopharyngeal carcinoma. Our results suggested that a set of potential targets for future investigation into the molecular mechanisms and biomarkers of nasopharyngeal carcinoma are identified by data mining and integration. To apply these gene expression profiles in clinical practice, it is necessary to improve the reliability and reproducibility of analysis model in independent datasets in the future. Nevertheless, our study provides an integrated bioinformatics analysis of DEGs and a group of useful targets for facilitating the early diagnosis and curative treatment of NPC. However, further molecular biological experiments are required to confirm the function of the identified genes in NPC.

## MATERIALS AND METHODS

### Microarray data

Two gene expression profiles (GSE12452 and GSE13597) were downloaded from GEO datasets (https://www.ncbi.nlm.nih.gov/geo/). GSE12452 was based on platform GPL570 (Affymetrix Human Genome U133 Plus 2.0 Array). Total RNA extracted from snap frozen laser-captured epithelium from 31 patients with Epstein-Barr virus positive undifferentiated nasopharyngeal carcinomas and 10 patients with no evidence of malignancy. mRNA expression levels were measured for essentially all human genes and all latent Epstein-Barr virus (EBV) genes in nasopharyngeal carcinoma tissue samples and normal nasopharyngeal tissues. Data were analyzed for differential gene expression between tumor and normal tissue and for correlations with levels of viral gene expression. GSE13597 which was based on platform GPL96 (Affymetrix Human Genome U133A Array) consisted of 25 EBV-related nasopharyngeal carcinoma samples and three normal control samples. Total RNA extracted from snap frozen nasopharyngeal biopsies from 25 patients with Epstein-Barr virus positive undifferentiated NPC and 3 patients with no evidence of malignancy.

#### Ethic statement

The authors declare that the procedures followed were in accordance with the regulations of the responsible Clinical Research Ethics Committee and in accordance with those of the World Medical Association and the Helsinki Declaration.

### Identification of DEGs

GEO2R (http://www.ncbi.nlm.nih.gov/geo/geo2r/) is a useful method to compare two or more groups of samples in order to identify genes that are differentially expressed across experimental conditions. We applied the adjusted P values (adj. P) to correct for the occurrence of false positive results using Benjamini and Hochberg false discovery rate method by default. We used log transformation to identify DEGs with a change twofold and defined a P value cut-off of <0.01 to be statistically significant.

### Gene ontology and pathway enrichment analysis of DGEs

The Database for Annotation, Visualization and Integrated Discovery (DAVID, http://david.abcc.ncifcrf.gov/) is a bioinformatics enrichment web tool for researchers to gain comprehensive high-throughput gene functional annotation analysis.[54] Gene ontology (GO) analysis and Kyoto Encyclopedia of Genes and Genomes (KEGG) pathway enrichment analysis were performed for analyzing DEGs at the functional level based on DAVID Bioinformatics Resources 6.8. P<0.05 was set as the cut-off criterion.

### Integration of protein–protein interaction (PPI) network and module selection

The Search Tool for the Retrieval of Interacting Genes (STRING) database (http://string-db.org) is an online program aimed to provide functional protein association networks. The newest STRING version 10.0 covers millions of proteins from more than 2000 organisms [55]. In order to provide context in molecular mechanism of cellular processing, we mapped protein-protein interaction (PPI) networks of DEGs by STRING and confidence score >0.4 was set as the cut-off criterion. Then, PPI networks were visualized using the Cytoscape software [56]. The APP Molecular Complex Detection (MCODE) was performed to screen modules of PPI network in Cytoscape_v3.4.0. The criteria were set as follows: MCODE scores >3 and number of nodes >4. Moreover, the function and pathway enrichment analysis were performed for DEGs in each modules by DAVID. P <0.05 was considered to have significant differences.
